# A Micromagnetic Protocol for Qualitatively Predicting Stochastic Domain Wall Pinning

**DOI:** 10.1038/s41598-017-17512-w

**Published:** 2017-12-19

**Authors:** K. A. Omari, T. J. Hayward

**Affiliations:** Department of Materials Science and Engineering, University of Sheffield, Sheffield, S10 2TN UK

## Abstract

Understanding dynamically-induced stochastic switching effects in soft ferromagnetic nanowires is a critical challenge for realising spintronic devices with deterministic switching behaviour. Here, we present a micromagnetic simulation protocol for qualitatively predicting dynamic stochastic domain wall (DW) pinning/depinning at artificial defect sites in Ni_80_Fe_20_ nanowires, and demonstrate its abilities by correlating its predictions with the results of focused magneto-optic Kerr effect measurements. We analyse DW pinning configurations in both thin nanowires (t = 10 nm) and thick nanowires (t = 40 nm) with both single (asymmetric) and double (symmetric) notches, showing how our approach provides understanding of the complex DW-defect interactions at the heart of stochastic pinning behaviours. Key results explained by our model include the total suppression of stochastic pinning at single notches in thick nanowires and the intrinsic stochasticity of pinning at double notches, despite their apparent insensitivity to DW chirality.

## Introduction

Understanding and controlling domain wall (DW) behaviour in ferromagnetic nanowires remain an essential field of research for realizing spintronic applications. Many proposed DW-based applications for information processing^1–3^ and memory^4,5^ rely on propagating DWs in nanowires with in-plane magnetisation (IPM) or perpendicular magnetic anisotropy (PMA) using external fields or spin polarized currents. Although recent research has focussed more on nanowires with PMA materials^6,7^, DWs in IPM nanowires remain interesting. This is because of the extra degree of freedom provided by DW chirality that can be utilized for information processing^8–10^ and in controlling switching trajectories in interconnected structures such as the Kagome spin-ice architecture^11,12^. DWs in IPM nanowires have also been proposed for use in more novel applications including conveying magnetic beads in biomedical applications^13^, and manipulating ultra-cold atoms^14,15^.

The propagation of DWs in nanowires can be controlled by creating localised energy potentials through introducing artificial defect sites^16,17^, or by locally changing the nanowires’ width/shape^18–20^. In case of artificial defects, the form of the energy potential at the pinning site depends on the interaction between the internal spin structure of the DW and the magnetic moments at the edge of the defect^17,21^. When a DW becomes pinned in such an energy potential, it can be overcome by the combination of applying a sufficiently large field/current and the effects of thermal activation^22^.

When DWs propagate above a critical field known as Walker Breakdown field (H_WB_) they undergo dynamic internal changes in magnetisation structure, known as Walker Breakdown (WB) transformations^23^. In the presence of edge roughness and thermal perturbations in real devices, an additional layer of stochasticity is added to the DW transformations and propagation^24^, such that, for consecutive switching events, DWs will be in different states at the same point in a nanowire, resulting in a range of pinned DW configurations at any given defect site. As the spin structures of each of these configurations will interact differently with a defect site, each may have a distinct depinning field. This yields highly stochastic multi-mode depinning field distributions^24–26^, a highly undesirable property for spintronic applications. In order to understand and control this dynamic stochasticity, it is important to understand the complex relationship between WB transformations, nanowire geometry, notch geometry and how the complex interaction of these defines stochastic pinning behaviours.

Although several studies^26,27^ have reported the effect of DW dynamics on DW-notch interactions and pinning/depinning behaviour, they have attributed stochastic pinning to the formation of four simple DW states, namely clockwise (CW) or anti-clockwise (ACW) vortex DWs (VDW) and upward or downward transverse DWs (TDW). Such attribution does not provide a complete picture of the stochastic pinning/depinning process; since, as we will show in this paper, each of these four basic DW types can appear in a variety of complex forms with different magnetisation configurations. Without a comprehensive understanding of these complex mechanisms a complete understanding of the nature of stochastic DW pinning behaviour cannot be achieved.

In this paper we present a new simulation protocol for understanding dynamically-induced stochastic DW pinning/depinning behaviours in magnetic nanowires with IPM. Our protocol allows us to study the full range of DW-defect interactions that may occur when DWs are undergoing Walker Breakdown transformations, and thus predict the full range of pinned DW states that may be formed. We then go on to qualitatively correlate the simulated behaviours with the results of single-reversal resolved focused magneto-optic Kerr effect measurements (FMOKE) of the same nanowire and defect geometries. Measurements were performed for two sets of nanowires with thicknesses of 10 nm and 40 nm, containing either single (asymmetric) or double (symmetric) notches of various depths. The geometry of the nanowires used throughout the study favours VDWs^28^.

We apply our simulation protocol to explain several complex features of stochastic DW pinning that are observed experimentally. Firstly we will explain the variation of stochastic pinning with nanowire thickness by showing how this changes the nature of a DW’s WB cycle, which in turn modifies the range of possible DW-notch interactions. Additionally, we will explain the counter-intuitive results that double notch defects, which are nominally insensitive to domain wall chirality, show complex stochastic pinning behaviours, while selected geometries of single notch defects, which are typically highly sensitive to DW chirality, show well-defined, quasi-deterministic depinning field distributions. Together, these results demonstrate the utility of our simulation protocol as a tool for understanding the complex magnetisation dynamics at the heart of stochastic DW pinning and depinning phenomena.

## Experimental and Simulation Methodology

**Figure 1:**
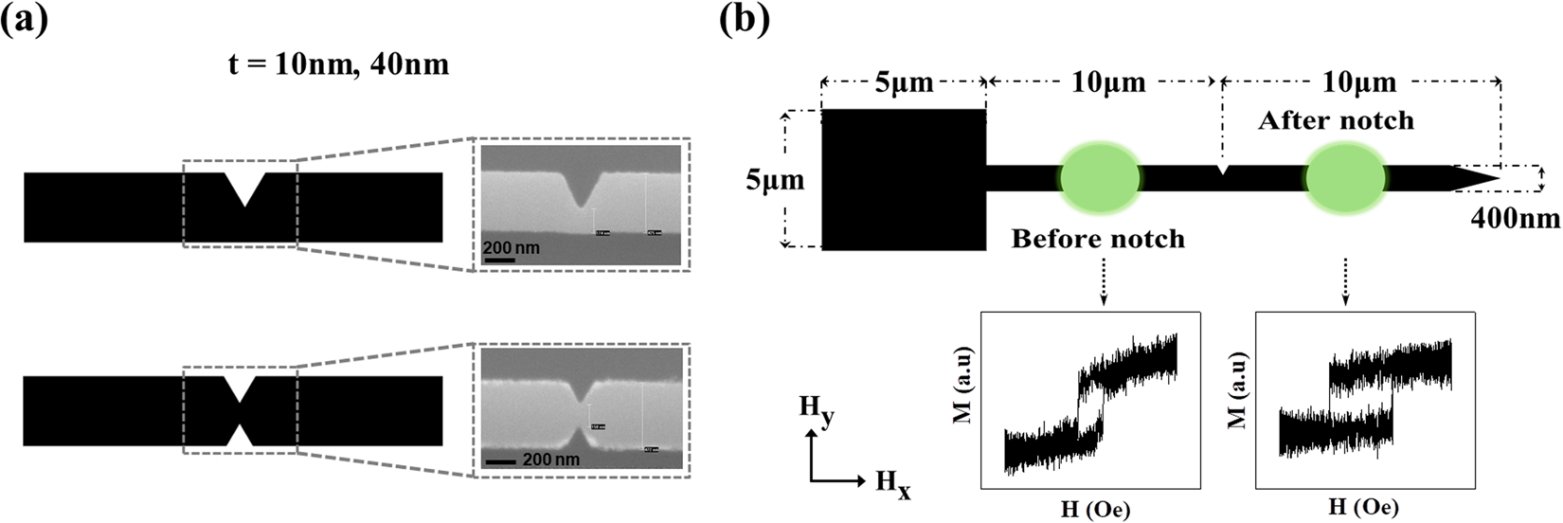
(**a**) Schematic diagram contrasting the geometries of the single and double notch designs. Inset showing SEM image of real single and double notch nanowires used in experiment for nanowire of t = 40nm and d_N_ = 0.7w and 0.35w, respectively. (**b**) Schematic diagram indicating the geometries of the experimentally measured nanowires. The green circles indicate the regions where the laser spot was focused in the MOKE measurements. Inset graphs show example single shot M/H curves for regions before and after notch for nanowire with t = 40nm and double notch with d_N_ = 0.35w.

Two sets of Ni_80_Fe_20_ nanowires, with thicknesses of t = 10 nm and t = 40 nm, widths w = 400 nm and lengths l = 20 μm were fabricated using electron-beam lithography, thermal evaporation and lift-off processing. Two different thicknesses of nanowire were studied so as to allow us to test the ability of the simulation protocol to provide understanding of stochastic pinning behaviours in nanowires that exhibit different WB transformation sequences, the details of which depend strongly on nanowire geometry^29–31^. Each nanowire was fabricated with a 5 × 5 μm^2^ pad on the left side of the nanowire to nucleate DWs^32^. The nanowires contained either a single or a double notch defect positioned at their midpoints (Fig. 1(a)). The depth of the single notches (d_N_) was varied from 15% of nanowire’s width (0.15w) to 0.7w; while for the double notches, d_N_ was varied from 0.15w to 0.35w. In all nanowires, the notch width was equal to d_N_. Readers may refer to Table ST1 in Supplementary Information for example SEM images of single and double notches of different depths in nanowire with t = 40 nm.

The nanowires’ switching behaviours were characterised using an FMOKE magnetometer, optimized to allow single-shot measurements. This capability was used to characterise the nanowires’ injection fields (H_inj_) and the depinning fields (H_d_) by focussing the laser spot in regions before and after the notch, respectively (Fig. 1(b)). Repeating these measurements over a total of 100 field cycles thus allowed measurement of the nanowires’ injection field distributions (IFD) and depinning field distributions (DFD). Readers may refer to Figures S1 and S2 in Supplementary Information for example single shot M/H curves obtained from FMOKE measurements of the type used to construct the IFD and DFD histograms.

To gain insight into dynamic DW-notch interactions in the nanowires characterised experimentally, micromagnetic simulations of DW pinning were performed using the MUMAX^3^ simulation package^33^. For each nanowire thickness and notch type (single or double notches) three different values of d_N_ were simulated, covering the range of d_N_ experimentally. All simulations were performed at T = 0 K.

**Figure 2:**
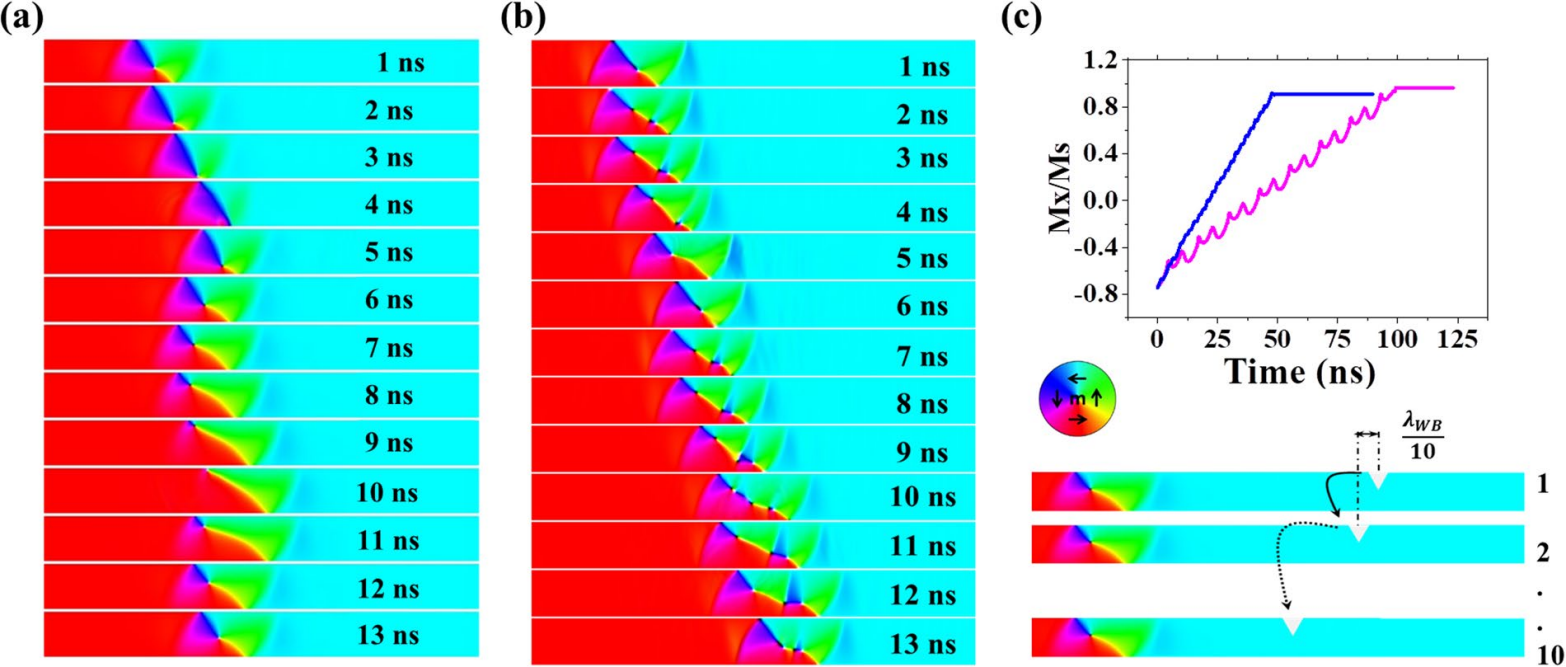
Simulation snapshots for DW propagation under WB transformation in isolated nanowires of t = 10nm at H_x_ = 30 Oe (**a**) and t = 40nm at H_x_ = 50 Oe (**b**). (**c**) (Top): Normalized magnetisation curve for t = 10nm (purple) and t = 40 nm (blue) nanowire showing WB cycle. (Bottom): Illustration of the basic approach taken by our modelling protocol. For each notch design 10 simulations were performed, with the position of the notch progressively shifted by one tenth of a WB period relative to the initial DW positions between the simulations.

Our simulation protocol for attempting to understand stochastic DW pinning aims to capture the full spectrum of DWs-notches interactions that can occur for a propagating DW. We achieved this as follows: First we simulated WB breakdown dynamics in a defect-free nanowire at a representative value of H_inj_ for t = 10 nm and t = 40 nm nanowires (Fig. 2), thus capturing the full range of states a DW passed through during its propagation. The distance travelled by the DW during one complete transformation cycle (𝜆_WB_) was then calculated. For t = 10 nm, WB period duration was equal to 13 ns and 𝜆_WB_ was calculated to be equal to 1050 nm. Whereas for t = 40 nm nanowire, period duration was equal to 5 ns and 𝜆_WB_ was deduced to be equal to 400 nm. We then performed 10 simulations in which the DW was propagated towards a notch, where the notches’ positions were shifted by 𝜆_WB_ /10 along the nanowire between simulations, thus allowing exploration of how the DWs interact with the notch at each point in its transformation cycle. When simulating nanowires containing single notches, simulations with both CW and ACW VDWs initial states were performed, to capture any chirality dependent interactions that occurred. For nanowires containing double notches, simulations were only performed for the ACW VDW chirality, due to the nanowires’ symmetry. To emulate the DFDs that would be expected from the nanowires, quasi-static simulations were performed to determine the depinning field of each pinned configuration. These switching fields were then used to derive DFDs where each discrete depinning mode was represented by a Gaussian distribution with amplitude equivalent to the number of times the pinned state was observed in the simulations, and with standard deviations = 1 Oe^34^.

## Results and Disicussion

### Nanowires of thickness t = 10 nm

**Figure 3:**
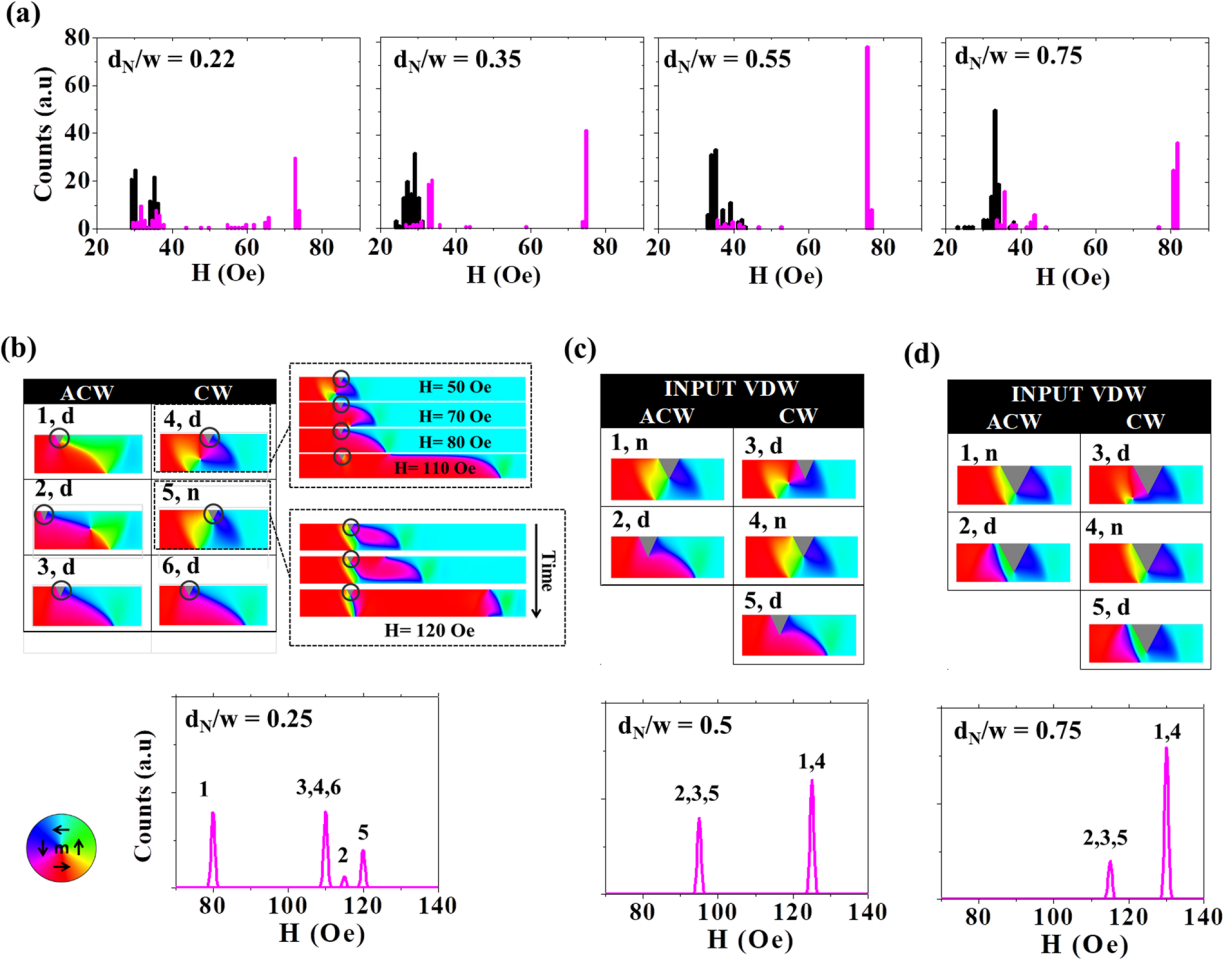
(**a**) Single-shot histograms of IFDs (black) and DFDs (purple) for nanowire with t = 10 nm and containing single notch defect. (**b**) Simulated DFDs and pinned DW configurations for single notches with d_N_ = 0.25w, (**c**) d_N_ = 0.5w (**d**) d_N_ = 0.75w at H_inj_ = 30 Oe. ‘d’ and ‘n’ label DW configurations that depinning via displacement and nucleation, respectively.

Figure 3(a) presents IFDs (black) and DFDs (purple) measured from t = 10 nm nanowires containing single notches with depths, d_N_ = 0.22w, 0.35w, 0.55w and 0.75w. All of the DFDs exhibited stochastic depinning behaviour, manifesting as multi-mode depinning field distributions and/or overlap between the IFDs and DFDs, indicating that DWs passed dynamically through defect sites without pinning in some reversals. Qualitative trends can be observed in the variation of the DFDs with notch size, with a wide distribution of depinning fields occurring for the smallest notches, contracting towards a dominating switching mode at 75 Oe − 85 Oe as the notch size increased. We note that these results contrast with that which would be expected from a simple model of DW pinning in these nanowires: VDWs are expected to be strongly favoured in nanowires of this geometry^28^, and have a chirality dependent interaction with single notch defect sites, leading to a splitting of the depinning fields^17^ for CW and ACW chiralities. Given that no attempts were made to control the chirality of the VDWs either at the point of nucleation or during propagation, a simple treatment of the problem would thus anticipate a DFD composed of two depinning modes with relatively even populations.

Simulations following the protocol described above were performed for notches with depths d_N_ = 0.25w, 0.5w 0.75w. DFDs derived from the simulation results (Fig. 3(b–d)) show qualitative similarities to the experimental data, in particular, a reduction in number of depinning modes and movement towards a dominant mode as d_N_ increases. We note that quantitative agreement between the measured and simulated DFDs was neither observed nor expected. For example, the simulated depinning fields are notably higher than the experimentally measured values, due to the absence of thermal activation effects in the simulations. Furthermore, contraction towards a dominant mode occurred more rapidly with notch size in the experimental data. Potential reasons for this will be discussed in detail below.

Figure 3(b–d) also present the pinned DW configurations observed for each of the notch geometries simulated. DWs pinned at the notches as both VDWs with CW and ACW chiralities, and as TDWs with UP and DOWN chiralities. This was expected since the WB sequence for this geometry showed periodic transformations between VDW and TDW structure (Fig. 2(a)). However, the formation of these pinned configurations did not necessarily lead to an equivalent number of peaks in the derived DFDs. For example, in some cases pinned DWs with the same basic DW type and chirality exhibited different depinning fields, while in other cases DWs with the very different structures depinned at the same field. For instance, the nanowire with d_N_ = 0.25w (Fig. 3(b)) exhibited four switching modes in the DFD. Two of these modes (80 Oe and 120 Oe) corresponded to two differently pinned TDW UP structures (configurations 1 and 5), while on the other hand, CW VDWs (configuration 4) and TDW DOWN structures (configurations 3 and 6) both exhibited the same depinning fields (110 Oe), due to the former evolving into the latter DW configuration during the depinning process.

An important factor in determining the depinning field of the DWs was whether depinning occurred by displacement of the existing DW structure or by nucleation of a new DW at the far side of notch (indicated by the letters ‘d’ and ‘n’ above the simulation snapshots in Fig. 3(b–d)). Example of simulation configurations illustrating these two switching mechanisms are shown in the inset of Fig. 3(b). Across all notch depths we only observed one DW configuration, UP TDWs positioned prior to the notch, that depinned via the nucleation mechanism. However, despite the minority of DW states for which nucleation occurred, the simulations showed this to become the dominant depinning mechanism as d_N_ increased, due to increased notch sizes favouring the formation of the UP TDW structures. For example, for the largest notch simulated (d_N_ = 0.75w) UP TDWs pinned in 80% of cases, compared to 17% in the simulations with the smallest notches (d_N_ = 0.25w). This is the origin of the movement towards a dominant switching mode as the notch size increases in the derived DFDs.

Collectively, the results from the simulation protocol offer substantial insight into the variations of the experimentally measured DFDs with notch size. For the smallest notch (d_N_ = 0.22w) we observed a wide DFD with many small peaks. Our simulations suggest this is because a wide range of pinned states could form, with a wide variety of depinning fields. As the notch size increased (d_N_ = 0.35w) the DFD collapsed into two clear peaks shown in the single-shot histogram, which our simulations indicated to represent the sub-populations of DW states that could be depinned by displacement  (ACW VDW, TDW DOWN), and those where nucleation at the notch was required for switching to proceed (TDW UP). Finally, for the largest notches characterised (d_N_ = 0.55w, 0.75w) the DFDs collapsed towards a single dominant mode. This trend was also observed in the simulations and was caused by DWs predominantly pinning in TDW UP configurations that required nucleation events to complete depinning. We note that the tendency towards a single mode appears to manifest more strongly in the experimental results than in the simulations. This may be attributed to two factors: Firstly, the simulation protocol explores the *full* range of possible DW-notch interactions, where as a real experiment may only probe a subset of these. Secondly, edge roughness of fabricated nanowires may tend to suppress true depinning mechanisms (as will be discussed further in relation to the 40 nm thick nanowires), and thus promote nucleation-based mechanisms.

**Figure 4:**
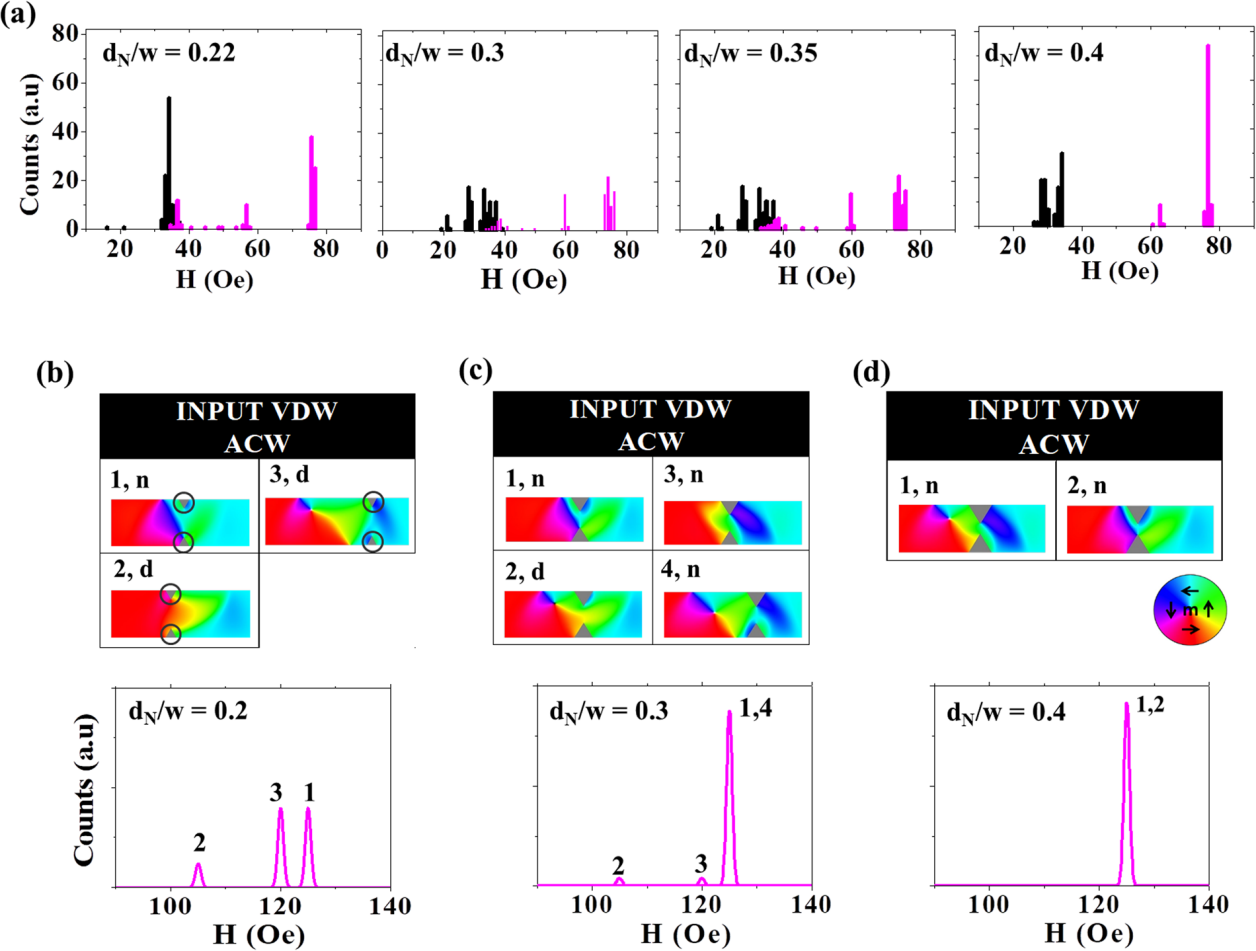
(**a**) Single-shot histograms of IFDs (black) and DFDs (purple) for nanowire with t = 10 nm containing double notch defects. (**b**) Simulated DFDs and pinned DW configurations for double notches with d_N_ = 0.2w (**c**) d_N_ = 0.3w (**d**) d_N_ = 0.4w at H_inj_ = 30 Oe. ‘d’ and ‘n’ label DW configurations that depinning via displacement and nucleation, respectively.

Figure 4 presents similar measurements and simulations for t = 10 nm nanowires containing double notches. The experimentally measured DFDs (Fig. 4(a)) showed a multi-mode switching behaviour for all d_N_; however, for the very highest values of d_N_ (0.4w), a movement towards a dominant mode can again be seen.

We note that, as for the single notches, the DFDs differ from what would be expected from simplistic model of DW pinning: For a nanowire that favours VDWs containing chirality insensitive double notches one would expect two pinned configurations, ACW and CW VDWs, both of which should have the same depinning field. Again, our simulation protocol provides understanding of why this wasn’t observed experimentally. Figure 4(b–d) present derived DFDs and pinned DW configurations for double notches with depths d_N_ = 0.2w, 0,3w and 0.4w. These results indicate that, like the single notches, the double notches could support both TDWs and VDWs, and that, even for these chirality insensitive defect sites, each of these basic configurations could pin in multiple ways (for examples of this consider the two TDWs configurations (1 & 2) seen at d_N_ = 0.2w (Fig. 4(b)), and the two VDWs configurations (2 & 4) seen at d_N_ = 0.3w (Fig. 4(c))). We attribute this behaviour to the sensitive nature of the DWs-double notch interaction, where the relaxation of the DW can be strongly affected by whether the leading edge of the DW interacts first with the spins near the top or near the bottom notch of the double notches, and how this eventually alters the DW transformations and interactions with the other notch.

We note that, once again, the number of possible pinned states does not always correlate with the number of modes observed in the derived DFDs, due to the fact that apparently very different DW configurations can share common depinning mechanisms and fields (e.g. configurations 1 and 2, Fig. 4(d)). Nevertheless, multiple depinning fields are predicted by simulations for d_N_ = 0.2w and 0.3w, before a collapse to a single nucleation-based mode occurs for d_N_ = 0.4w. This mirrors the qualitative trends observed in the experimental data, and allows us to understand their origins.

### Nanowires of thickness t = 40 nm

**Figure 5:**
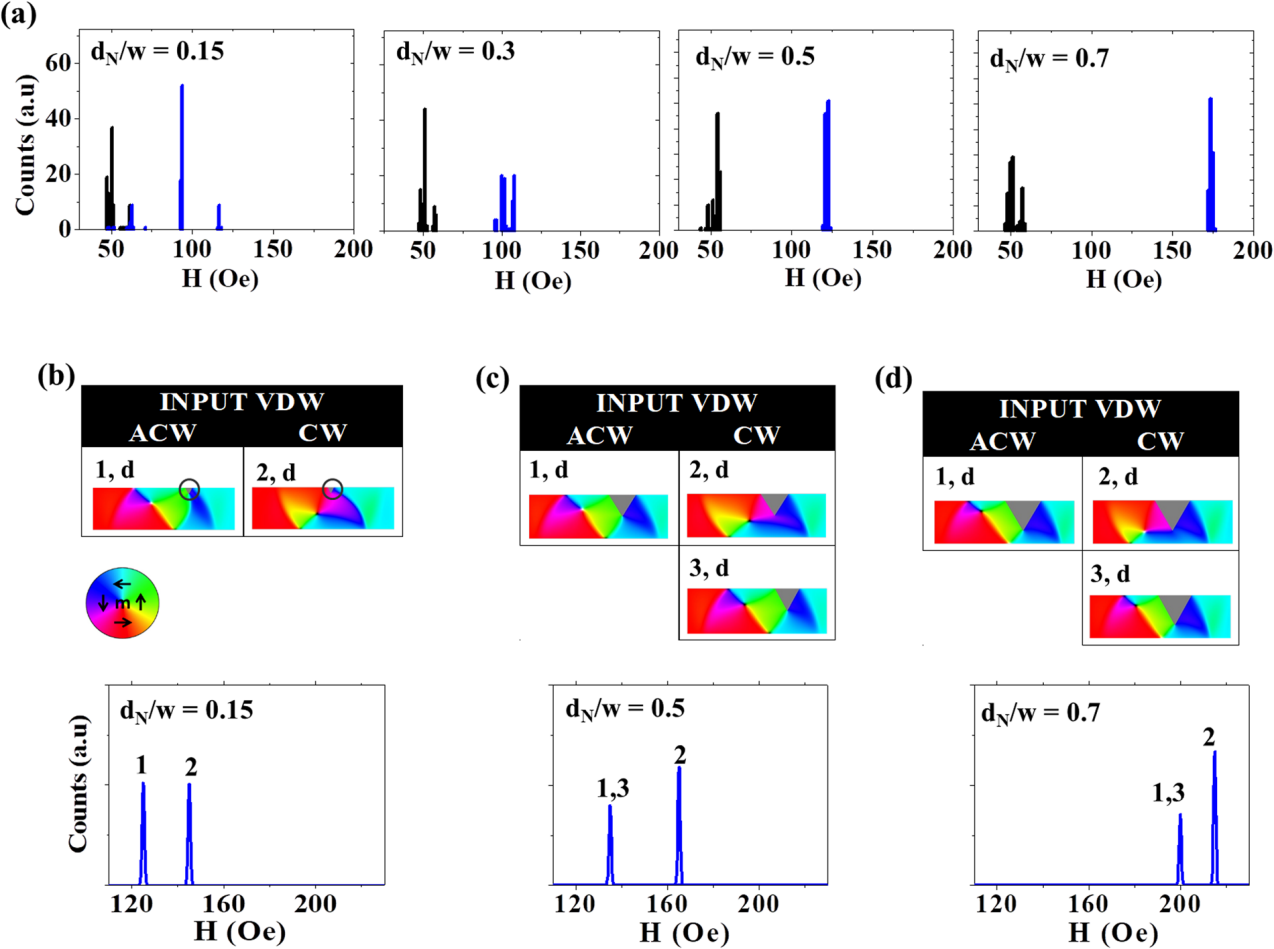
(**a**) Single-shot histograms of IFDs (black) and DFDs (blue) for nanowire with t = 40 nm containing single notch defect. (**b**) Simulated DFDs and pinned DW configurations for double notches with d_N_ = 0.15w (**c**) d_N_ = 0.5w (**d**) d_N_ = 0.7w at H_inj_ = 50 Oe. ‘d’ and ‘n’ label DW configurations that depinning via displacement and nucleation, respectively.

Figure 5 presents measurements and simulations for t = 40 nm nanowires containing single notch defects. The large thickness of these nanowires meant that they were expected to favour VDWs over TDWs even more strongly than the t = 10 nm nanowires. It also meant that they were expected to produce very different WB dynamics, and thus DW-notch interactions, than their thinner counterparts, as may be seen to be the case in Fig. 2. These differences provided an opportunity to examine the robustness of our simulation protocol.

Figure 5(a) presents IFDs (black) and DFDs (blue) obtained from measurements of nanowires containing single notches with depths d_N_ = 0.15w, 0.3w, 0.5w and 0.7w. These data revealed a very clear trend with bimodal DFDs at lower d_N_ (d_N_ = 0.15w, 0.3w) converging into quasi-deterministic single-mode DFDs for d_N_ ≥ 0.5w. This is again a surprising result as one would anticipate that a chirality sensitive single notch defect would differentiate the depinning fields of ACW and CW VDWs, thus producing bimodal DFDs for all defect sizes.

Figure 5(b–d) present derived DFDs and pinned DW configurations simulated for notch depths of d_N_ = 0.15w, 0.5w and 0.7w. For the first time, substantial differences can be seen in the qualitative trends exhibited by the experimental data and the DFDs derived from the simulations, with the simulation results exhibiting bimodal DFDs for all values of d_N_. Nevertheless, a bias towards the depinning mode with highest H_d_ was observed as the notch depth increased, with this mode being favoured by a factor 2:1 at the largest notch depths.

The pinning configurations indicated in Fig. 5(b–d) show that in the t = 40 nm nanowires only VDWs pinned, unlike in the t = 10 nm nanowires where a mixture of VDWs and TDWs pinned. The differences in pinned DW configurations between the two thicknesses can be attributed to two factors. Firstly, as previously mentioned, VDWs have much lower energies than TDWs in nanowires of these thicknesses. Secondly, as we have previously shown in [10], WB transformations in nanowires of this geometry have a slightly unusual form where the DWs undergo elongations and contractions of their basic VDW structures while preserving their overall chirality (also indicated in Fig. 2(b)). As a result, TDW configurations are unlikely to be incident on the notch. Consequently, the pinning configurations are limited to the VDW shape with its two chiralities providing the two distinct modes in the derived DFDs.

**Figure 6:**
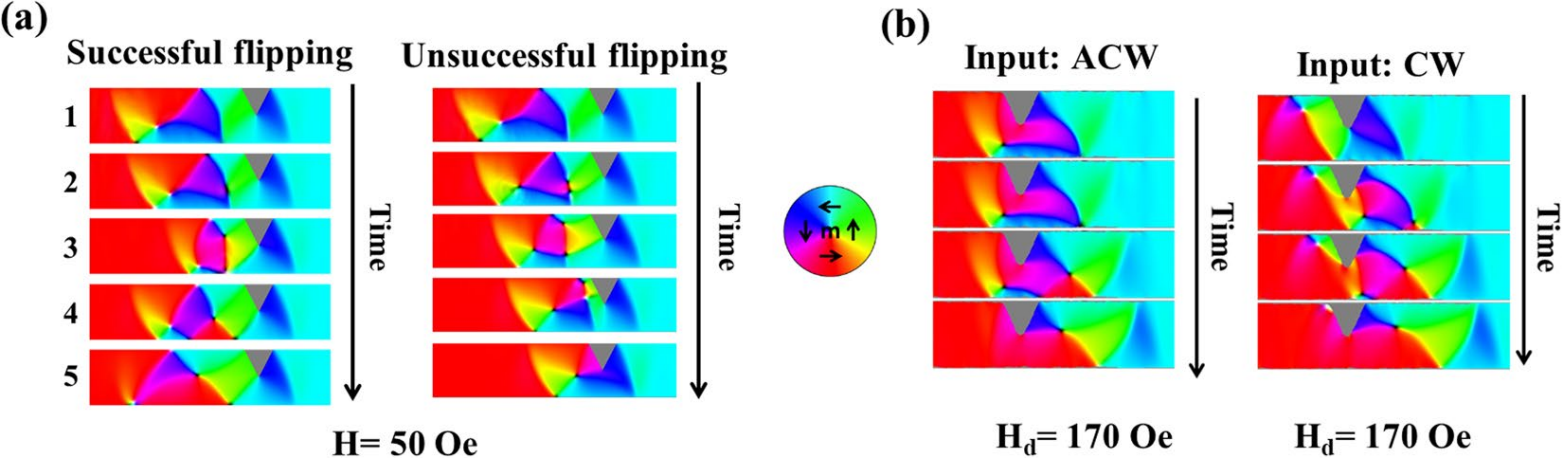
(**a**) Simulation snapshots contrasting the successful flipping of the chirality of a CW VDW (left) to a case where flipping was unsuccessful (right) in nanowires with t = 40 nm and d_N_ = 0.5w. H_inj_ = 50 Oe. (**b**) Simulation snapshots of DW depinning in a rough nanowire with a single notch of d_N_ = 0.5w. Both ACW and CW VDWs depin via a nucleation mechanism with H_d_ = 170 Oe.

The discrepancies between the experimental DFDs and those predicted by the simulation protocol can be explained by two phenomena. Firstly, in cases where a CW VDW was incident on the notches we noticed several simulations where their chiralities flipped to ACW, but no examples where the opposite process took place. Flipping occurred when the downward-oriented spins of the leading edge of the CW DW interacted with upward-orientated spins at the edge of the notch (Figs 6(a)–2). This region of magnetic frustration resulted in the nucleation of a new vortex core with ACW chirality to lower its exchange energy. If the new DW core was nucleated in a manner that allowed its vortex to expand before being pushed to the top edge of the nanowire then the newly nucleated DW formed with a flipped chirality. On the other hand, if the new vortex core was nucleated at close to the notch and top edge of the nanowire, the new vortex annihilated as it was pushed towards the top edge of the notch yielding unsuccessful flipping (refer to Fig. 6(a)–4 in the Unsuccessful flipping case). Simulation results showed successful flipping occurring 20% of the time for d_N_ = 0.5w and 30% of the time at d_N_ = 0.7 (compared to 0% flipping in nanowires with notch depth of 0.15w). Chirality flipping following a different flipping mechanism was reported by Brando *et al* ^35^.

The CW chirality flipping observed in the simulations explains why the derived DFDs showed increasingly asymmetric populations of the two depinning modes as the notch size increased. However, the frequency of this process occurring was 30% at most in the simulations, and thus it could not provide clear justification for the single-model behaviours seen experimentally, unless it is assumed that, coincidently, our measurements only subsampled DW-notch interactions where flipping was likely to be successful. As this seemed unlikely we also considered a second explanation: that edge roughness in the experimentally measured nanowires forced the depinning processes of the two pinned VDW states to converge towards a single mechanism, and thus depinning field. To explore this, simulations of a nanowire (d_N_ = 0.5w) with realistic edge roughness were performed. In these simulations the nanowires edge profile was imported from an SEM image of the nanowire measured experimentally.

Figure 6(b) illustrates the depinning mechanisms observed for ACW and CW VDWs pinned at the notch in the roughened nanowire. In both cases switching occurred at H_d_ = 170 Oe via the nucleation of a ACW VDW on the left-hand side of the notch. This suggests that, for the larger notches measured experimentally, the effects of edge roughness were sufficient to force depinning to proceed via a nucleation-based mechanism for all pinned DW configurations, thus producing quasi-deterministic, single-mode DFDs. Further evidence supporting this explanation can be seen in the experimentally measured DFD for d_N_ = 0.3w, where a bi-modal DFD was observed, but with a much smaller separation between modes than was observed in the simulated DFDs. Here, it is likely measurements were performed at the cusp of edge roughness forcing the DFDs to collapse to single mode character.

**Figure 7:**
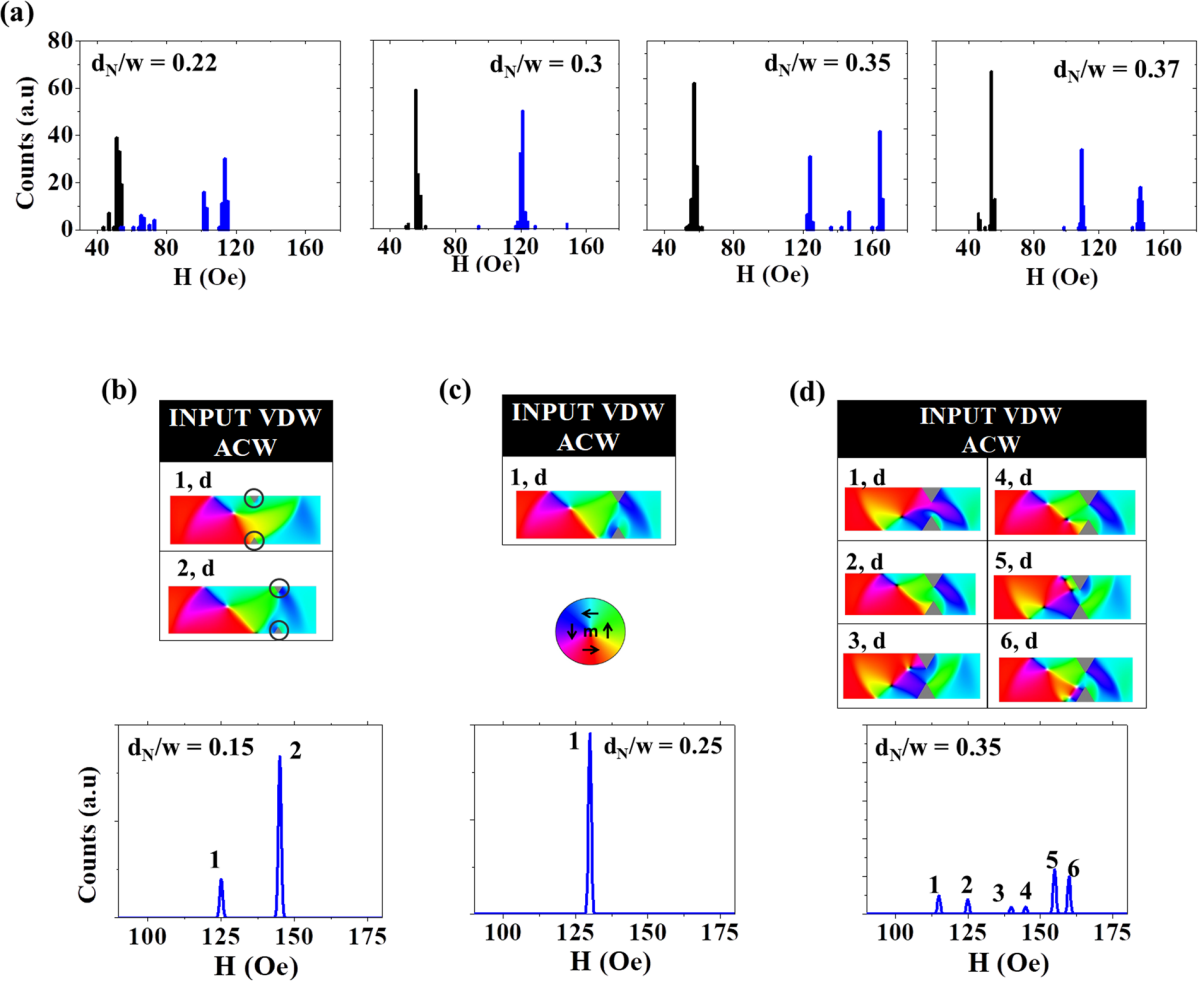
(**a**) Single-shot histograms of IFD (black) and DFD (blue) for nanowire with t = 40 nm containing double notch defect. (**b**) Simulated DFDs and pinned DW configurations for double notches with d_N_ = 0.15w (**c**) d_N_ = 0.25w (**d**) d_N_ = 0.35w at H_inj_ = 50 Oe. ‘d’ and ‘n’ label DW configurations that depinning via displacement and nucleation, respectively.

Figure 7 illustrates a final set of measurements and simulations, performed for t = 40 nm nanowires containing double notches. Given the strong bias towards pinning VDWs in t = 40 nm it would be expected that these chirality-insensitive defect sites would show single mode DFDs. However, once again the experimentally measured DFDs (Fig. 7(a)), confound this expectation, exhibiting multimode DFDs at both extremities of the range of measured notch sizes (d_N_ = 0.22w, 0.35w, 0.37w), with a single mode DFD only being observed for an intermediate notch depth of d_N_ = 0.30.

Remarkably, the DFDs derived from simulations with similarly sized notches (d_N_ = 0.15, 0.25, 0.35) appeared to, at least qualitatively, reproduce this trend, with multi-mode DFDs being produced for d_N_ = 0.15w and 0.35w, and a single mode DFD being observed at the intermediate notch depth, d_N_ = 0.25w (Fig. 7(b)–(d)). Analysis of the pinned states showed that the multimode DFDs resulted from VDWs pinning either in slightly different locations relative to the notch (e.g. pinned states 1 & 2, Fig. 7(b)), or with complex multi-vortex perturbations of their basic magnetisation structure (e.g. pinned states 3, 4, 5 & 6, Fig. 7(d)). In the simulations, these slight variations in the configurations of the pinned VDWs were sufficient to cause significant variations in the VDWs depinning fields, although the experimental DFDs for larger notches (e.g. d_N_ = 0.35w) were noticeably simpler than those in equivalent simulations (Fig. 7(c)), perhaps suggesting that edge roughness was again capable of homogenising depinning mechanisms of different pinned states. Our simulations suggest that the collapse of the DFDs to a single mode for the intermediate notch sizes occurred because, for these values of d_N_, DW-notch interactions tend to only form a single, well-defined pinned DW structure, thus producing a single well-defined depinning field.

## Conclusions

In this paper we have described a micromagnetic simulation protocol that allowed us to qualitatively understand the stochastic pinning behaviour of domain walls in soft ferromagnetic nanowires. We then verified the capabilities of the model by comparing its predictions with focused MOKE measurements of DW pinning in nanowires containing both single and double notches of a variety of sizes.

The robustness of the simulation protocol was demonstrated by its ability to predict and assist interpretation of qualitative trends in the form of DFDs across a large range of defect geometries for two thicknesses of nanowire (t = 10 nm and t = 40 nm). Furthermore, in the one case where the simulation results differed significantly from those obtained experimentally (t = 40 nm, double notches), agreement was restored by improving the accuracy of our description of the simulated system, in this case by adding edge roughness. We note that further evidence of the protocols capabilities may be seen in^36^, where it is used to successfully predict behaviour in a further nanowire system (t = 25 nm) with differing WB behaviours and DW energetics.

There are however clear limitations to the protocols abilities. While it is able to predict broad qualitative trends it is unable to make quantitative predictions. For example, it cannot precisely predict DW depinning fields, the populations of DFD modes or the exact notch depth at which critical transitions in the forms of DFDs occur (e.g from bi-modal to single mode behaviour). Some of these discrepancies might be improved upon by refinement of description of the simulated system (e.g. notch shape, edge profile, precise magnetic properties), but it is unlikely exact agreement could ever be achieved. This is primarily due to two factors: Firstly, our protocol neglects the influence of thermal fluctuations, which have a strong effect on both DW depinning^37^ and propagation^21^. Secondly, our protocol equally samples all DW-notch interactions available for a freely propagating DW, however a given experiment may sub-sample a population of these or introduce other forms of interactions through distortion of the DWs’ magnetisation profiles during their injection processes^36^.

Despite the above limitations our protocol provides deep insight into some counter-intuitive phenomena observed experimentally. For examples, double notches seem to be intrinsically stochastic for both thicknesses studied and for most values of d_N_, despite their chirality insensitive nature. Our simulations show that this is because the complex energy landscape of the notches can stabilise a variety of perturbations on standard DW configurations, each with their own distinct depinning fields. Conversely, for single notches, which would be expected to produce multi-mode DFDs due to their chirality sensitive nature, we observe a convergence to or towards a quasi-deterministic single mode DFD at larger notch depths. Our simulations show this occurs because, for large notches, DWs pinned with disparate structures, tend towards depinning via a single, nucleation-based mechanism.

When combined together our results demonstrate the functionality of a new tool that could be used to assist the development of IPM nanowires with deterministic switching behaviour for future spintronic applications.

## Methods

Nanowires with a nominal composition of Ni_80_Fe_20_ were fabricated by electron beam lithography with lift-off processing. Metallisation was performed by thermal evaporation at a base pressure of 1 × 10^−7^ mbar.

The switching behaviours of the nanowires were characterised using an FMOKE magnetometer with a ~5 μm laser spot. During measurement, magnetic fields with amplitude ± 600 Oe and frequency 27 Hz were applied along the length of the nanowires to saturate and switch them. The system was optimised for single-shot measurements of the nanowires magnetic switching, so as to allow the building of IFDs and DFDs by custom built analysis software.

Micromagnetic simulations were performed using the MUMAX^3^ simulation package^33^. The simulated nanowires had same width and thickness as nanowires characterized using MOKE, but lengths of l = 6 μm, with the effects of the poles at the nanowires ends being subtracted using built-in functions of the simulation package. We used standard values for the material parameters of Ni_80_Fe_20_: saturation magnetisation, M_s_ = 860 kA/m, exchange stiffness A_ex_ = 13 pJ/m and anisotropy constant K = 0 J/m^3^. When performing dynamic simulations of DWs propagating to and pinning at notches a realistic damping constant of α = 0.02 was used, while an artificially high value of α = 1 was used in quasi-static simulations performed to determine the depinning fields of pinned domain walls. We used mesh sizes of 2.5 × 2.5 × 5 nm^3^ for the t = 10 nm nanowires and 2.5 × 2.5 × 10 nm^3^ t = 40 nm nanowires. These meshes produced dynamics that were found to be consistent with those observed for an isotropic 2.5 × 2.5 × 2.5 nm^3^ meshes.

## Electronic supplementary material

Supplementary Information
