# Supplementary material for: A Micromagnetic Protocol for Qualitatively Predicting Stochastic Domain Wall Pinning
Source: Sci Rep. 2017 Dec 19;7:17862. doi: 10.1038/s41598-017-17512-w (PMC5736692; doi:10.1038/s41598-017-17512-w)
Supplement: Supplementary file 1 — Supplementary Information [file 41598_2017_17512_MOESM1_ESM.pdf]

Supplementary Information

A Micromagnetic Protocol for Qualitatively Predicting Stochastic Domain Wall Pinning

K.A. Omari and T.J. Hayward*

Department of Materials Science and Engineering, University of Sheffield, UK

*email: t.hayward@sheffield.ac.uk

1. SEM images of single and double notches for nanowire of t=40 nm

Supplementary Table ST1

Double Notch	Notch depth	Single Notch	Notch depth
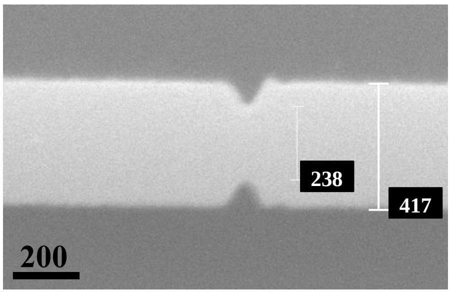	0.22	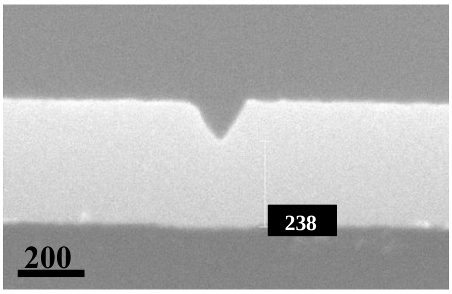	0.15
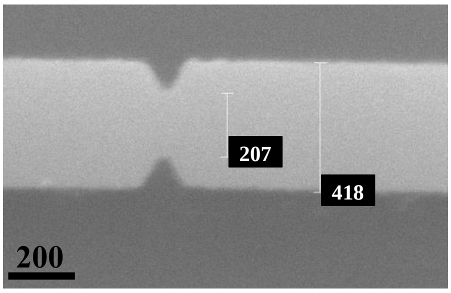	0.25	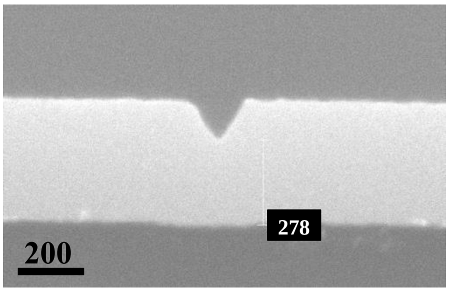	0.30
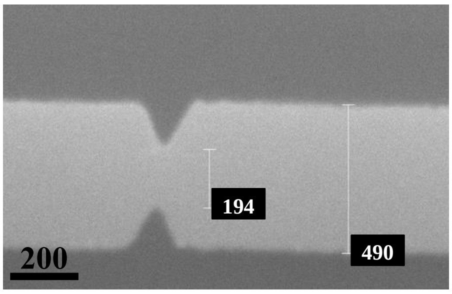	0.30	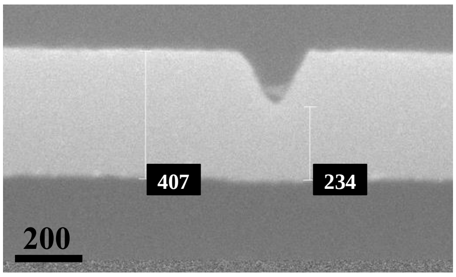	0.50
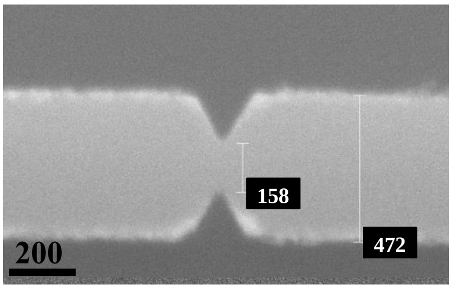	0.35	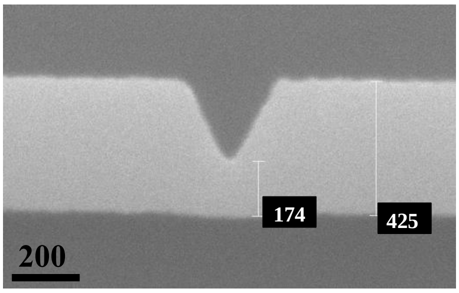	0.70

*All dimensions are in nm

Supplementary table ST1 shows the SEM images of the set of single and double notches used for the measurement of nanowires with t= 40 nm. Images were taken using the secondary electron detector under 10 keV electron-gun tension.

The nanowire width and gap of each image were measured using the SEM measuring tool in the imaging software. The fractional 'notch depth' was then deduced using by the following formulas:

$$\textbf{Single notch nanowire: } \frac{\textbf{Gap width} - \textbf{Nanowire width}}{\textbf{Nanowire width}}$$

$$\textbf{Double notch nanowire: } \frac{1}{2}\,\frac{\textbf{Gap width} - \textbf{Nanowire width}}{\textbf{Nanowire width}}$$

2. Measurement data for nanowire of t=40nm and double notch with d_N = 0.35w

Figure S1 and S2 below present the raw data of the switching field distribution obtained from single-shot measurements of M/H curves generated from the FMOKE setup. The M/H curves of the single-shot measurements in the figures (presented as insets in in dashed frames) were correlated with the switching states presented in the injection field distribution (IFD) (Figure S1) and depinning field distribution (DFD) (Figure S2) histograms obtained for region before and after the double notch, respectively. Numbers above the histogram bar and in each set of insets indicate the switching distribution/states. Such data presentation provides an insight into the robustness of our measurements and the signal-to-noise ratio.

The IFD and DFD histograms are an enlargement of the histogram presented in Figure 7(a) of the manuscript for nanowire of d_N=0.35w.

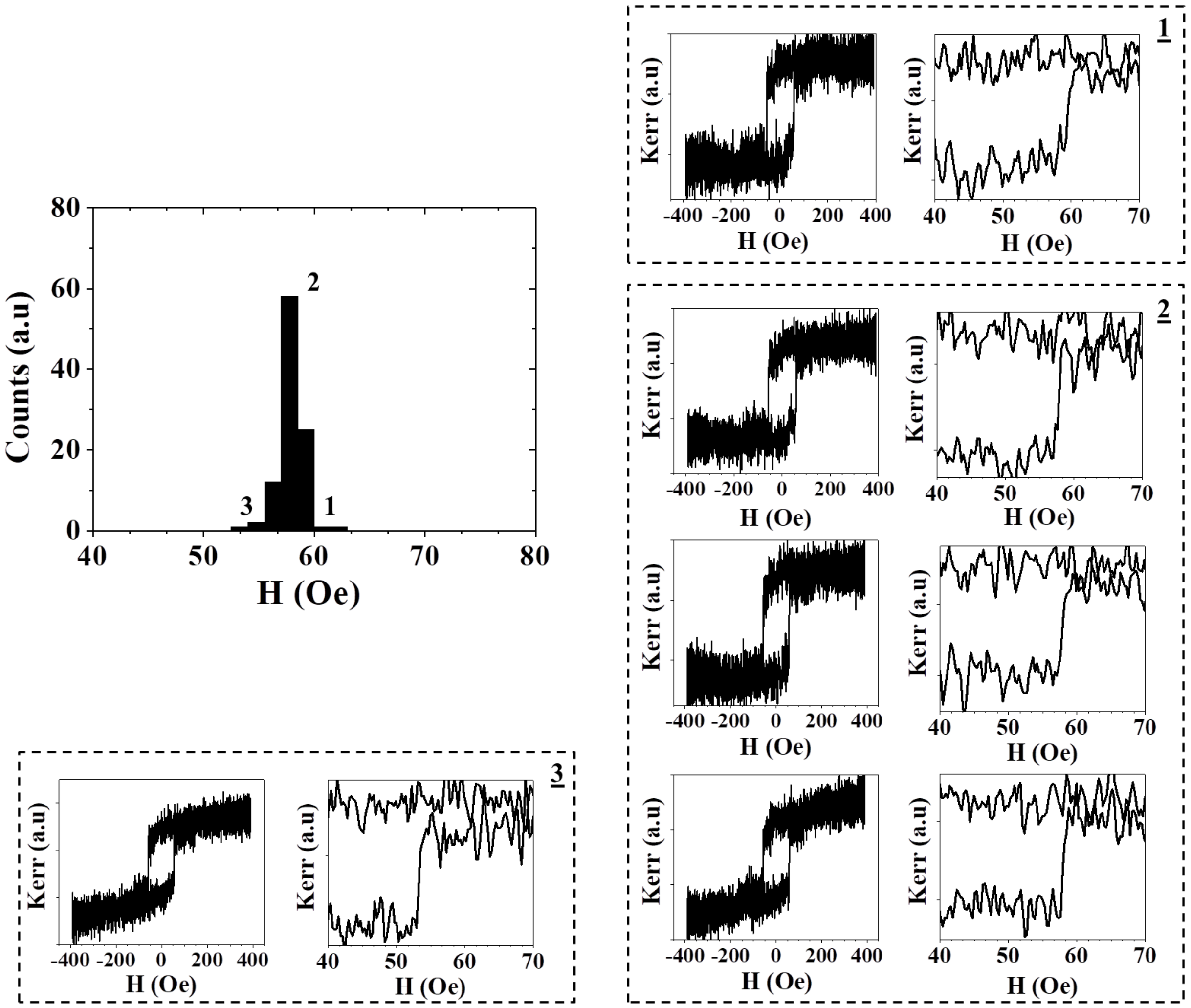

Figure S1: IFD histogram showing the 100 single-shot switching field measurements for region before the double notch for nanowire of t=40 nm with double notch of d_N= 0.35w. Numbers indicate different switching fields (bar bin size =1.5 Oe). Insets in dashed frame show samples of single-shot measurements at each different switching state along with enlarged section of switching instant.

The IFD indicated a well-defined injection process with a Gaussian-like distribution where majority of injection instances occurred at H = 58 Oe ± 0.75. Such distribution shape suggests that injection is following a single switching route that experienced thermal activation.

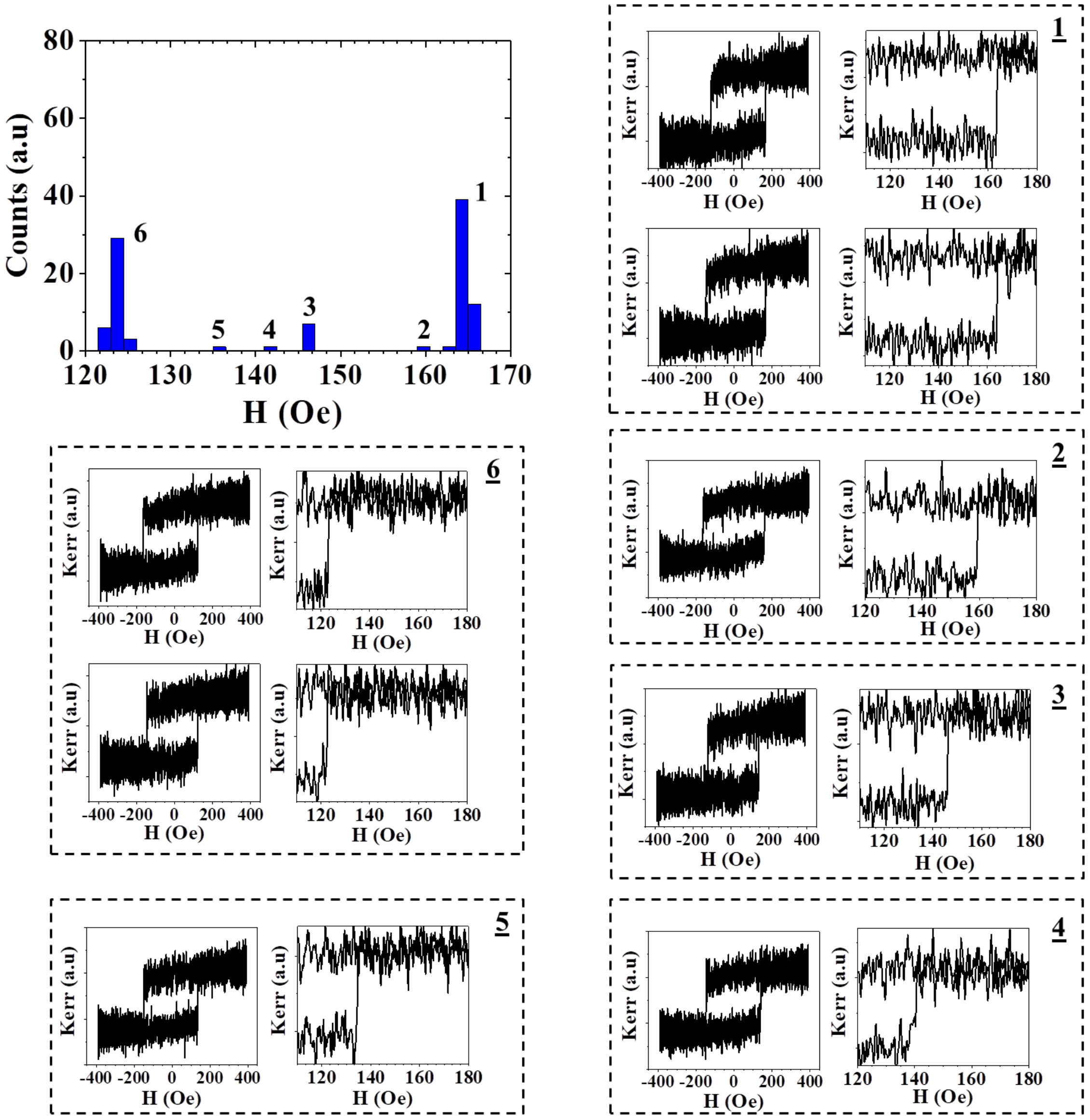

Figure S2: DFD histogram showing the 100 single-shot switching field measurements for region after the double notch for nanowire of t=40 nm with double notch of d_N= 0.35w (bar bin size =1.5 Oe). Numbers indicate different switching states. Insets in dashed frame show samples of single-shot measurements at each different switching state along with enlarged section of switching instant.

The DFD distribution shape indicated a multi-state switching process with multi-peak distributions. This can be attributed to dynamic stochasticity along with thermal activation that trigger changes in domain wall structure during its motion and relaxation (interaction with notch). This is expected to result in different multi-mode switching behaviour (different switching routes) as explained in the manuscript. Two dominating switching states are state 1 and state 6 where majority of switching in those states occur at H= 123 Oe ± 0.75 and 164 ± 0.75, respectively.
